# Do pastoral and agro-pastoral perceptions align with observed climate extremes? Evidence from the Koh-e-Suleiman Range, Pakistan

**DOI:** 10.1038/s41598-026-41100-6

**Published:** 2026-03-05

**Authors:** Waqar Ul Hassan Tareen, Eva Schlecht

**Affiliations:** Animal Husbandry in the Tropics and Subtropics, University of Kassel and Georg-August-Universität Göttingen, Albrecht-Thaer-Weg 3, 37075 Göttingen, Germany

**Keywords:** Climate perception, Observed climate trends, Pastoralists and agro-pastoralists, Climate sciences, Environmental sciences, Environmental social sciences

## Abstract

This study examined the relationship between climate perceptions and observed trends among pastoralist and agro-pastoralist communities in the Koh-e-Suleiman Range, Pakistan. Household perception data were collected from 198 respondents and analyzed alongside climatic records across two time scales (1980–2022; 2013–2022). Data from the Pakistan Meteorological Department were used to compute 29 extreme climate indices, and trends were assessed using the Mann–Kendall and Sen’s slope tests. Perceptions of seven climate variables were compared with observed trends through accuracy tests, bias classification, regression, and machine-learning models. Perceptions aligned closely with observed trends for floods, rain intensity, temperature, and warm spells ($\ge$ 80% accuracy), and moderately for cold spells (71.7%) and rainfall (60.6%). Perceptions of drought spells were predominantly inaccurate, with 75.3% of respondents overestimating their occurrence. Regression analyses identified education, age, and livestock ownership as associated with perception accuracy. Classification and Regression Tree (CART) machine learning analysis, in contrast, revealed non-linear effects: income, age, and livestock herd size shaped drought spell perception, while livestock numbers and age influenced rainfall perception. These findings highlight the value of integrating observed climate extremes with local perceptions to better understand perception observation alignment and inform context- sensitive climate risk communication in data-scarce pastoral regions.

## Introduction

Climate change is a global phenomenon that manifests distinctly at regional and local scales, significantly impacting ecosystems, livelihoods, and economic stability of societies around the world. People living in mountainous, arid and semi-arid regions are particularly affected^1^. The IPCC concludes that climate change has already caused widespread harms to people and nature, and that risks to ecosystems, food security, and human health escalate with each increment of warming^2^, with rising temperatures and more intense heatwaves threatening food production, health and livelihoods of people, and adding pressure on infrastructure and governance^3^. Climate projections for Asia forecast more frequent and severe heatwaves, prolonged droughts, intensified monsoon floods, and accelerated glacier melt^4^.

Pakistan is ranked among the world’s most climate-affected countries, with the Climate Risk Index 2025 identifying it as the most impacted country in 2022 due to unprecedented flooding and substantial socio-economic losses^5^. Within this broader context of climate vulnerability, the Koh-e-Suleiman Range, an extension of the Hindu Kush that links Balochistan to Pakistan’s other provinces, hosts Baloch tribal communities whose livelihoods are closely tied to climate-sensitive systems. Local pastoralist and agro-pastoralist households rely almost entirely on livestock rearing and seasonal grazing, drawing on traditional land and resource-management practices that both buffer against and are challenged by rainfall variability, temperature extremes, and hydrological shocks^6^.

Local perceptions of climate change are central to shaping adaptation strategies, influencing how risks are prioritized and resources are allocated across households and seasons for climate-resilience^7–9^. However, these perceptions may at times diverge from empirical climate records, which can lead to maladaptive decisions^10^. Such divergences are often shaped by lived experience, understanding why and how these discrepancies occur is essential for designing effective, evidence-based resilience strategies that strengthen community responses to environmental change^11^.

Research on pastoralist communities are historically underrepresented in climate perception literature. Pastoralists across Africa and Asia consistently report rising temperatures, shifts in grazing cycles, and dwindling water resources as immediate threats to their herds and livelihoods^12,13^. These observations are often grounded in traditional ecological knowledge, built through generations of close interaction with their environment, and in many cases align with meteorological trends. Geographic context and socio-demographic factors strongly shape climate change perceptions^14–16^. Key socio-demographic determinants include age, gender, education, family size, asset ownership, occupation, communication networks, and prior knowledge. At the same time, among pastoralists, perceptions are particularly influenced by the household head’s gender, livestock ownership, herd size, and access to extension services^13,17^.

Comparing local perceptions with systematically collected climate data is a challenging endeavor, as it tests both the validity of subjective observations and their implications for adaptation behavior^12,18^. Accurately quantifying climate-change perceptions and identifying their underlying socio-economic determinants requires a robust methodological framework^19^.

Despite growing interest in climate perception research, most studies in Pakistan have focused on crop-farming households^20–24^. Several farm-level studies in the country have documented how rural households perceive temperature increases and rainfall variability, often linking these perceptions to adaptation practices^20,25^. Studies in mountainous regions of northern Pakistan further highlight how local communities perceive climate change impacts in high-altitude environments^21^. More recently, perception–observation comparisons in the Upper Indus Basin have examined local experiences of climate variability alongside instrumental records^26^, while community-based studies in Khyber Pakhtunkhwa emphasized the role of local knowledge in shaping climate adaptation behaviors^22^. In parallel, much of the broader literature from Pakistan and South Asia has emphasized climate projections^27^, compound climate indices^28^, regional heat stress diagnostics^29^, or crop-yield responses to drought indicators^30^, rather than examining how observed climate extremes are perceived and interpreted by local communities. Yet, the mentioned studies largely overlooked pastoralist and agro-pastoralist communities, resulting in a significant knowledge gap.

Against this gap, the present study advances climate perception research by adopting a robust and integrative methodological framework that explicitly addresses under-researched domains. It (1) specifically examines pastoral and agro-pastoral livelihoods, rather than crop-farming systems that dominate most climate perception studies in Pakistan and South Asia, thereby addressing an important and underrepresented population group. It (2) Uses ETCCDI extreme climate indices to analyse long-term and short-term changes in temperature and precipitation. This enables an assessment of climate extremes rather than mean climate variables and provides a standardized framework for evaluating observed climate variability. It (3) quantifies perception accuracy and bias by systematically comparing community reported climate perceptions with observed meteorological trends. In doing so, the study moves beyond a mere documentation of perceptions. (4) By combining conventional logistic regression models with Classification and Regression Tree (CART) analysis, the study identifies both linear and non-linear socio-economic drivers of climate perception accuracy and bias, offering deeper insight into threshold effects and interaction structures.

By integrating these four complementary analytical approaches, the present study provides a comprehensive and methodologically rigorous assessment of climate perception–observation alignment among pastoral and agro-pastoral households.

## Methods

### Description of study area

**Fig. 1:**
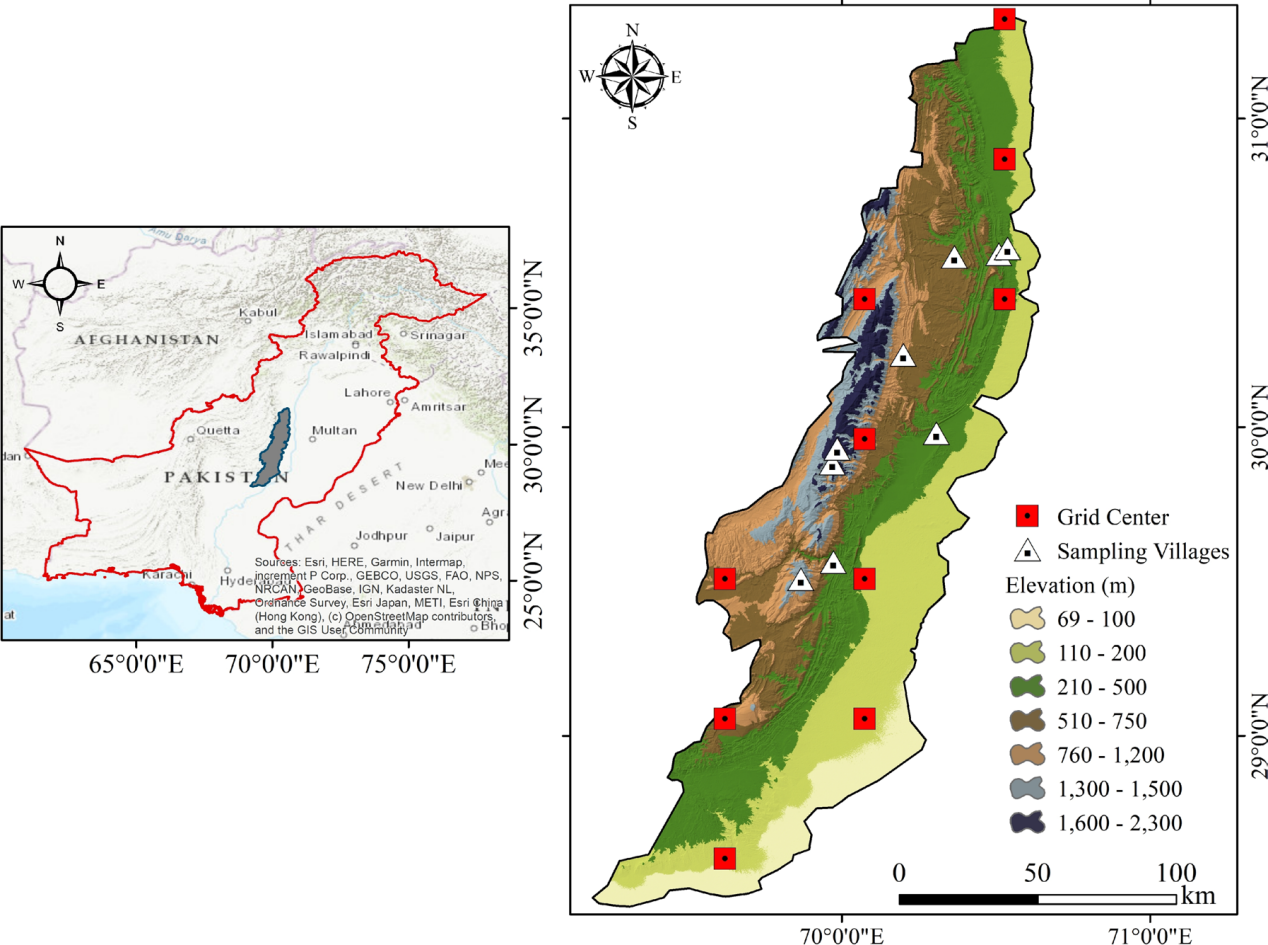
Map of the study area in the Koh-e-Suleiman region, Punjab, Pakistan, covering the tribal regions of Dera Ghazi Khan and Rajanpur in southwestern Punjab (right side). The left inset provides the national context within Pakistan. Sampling sites (white triangles) and climate data grid centers (red squares) are overlaid on an elevation map, ranging from 69 m to >2300 m above sea level. The map was drawn in ArcGIS Desktop 10.5 (https://www.esri.com/).

The study was conducted in the Punjab tribal belt of the Koh-e-Suleiman Range, central Pakistan, covering the tribal territories of Dera Ghazi Khan and Rajanpur districts (see the details in Fig. 1). This north–south oriented mountain system extends approximately 450 km, covering an area of 11,488 km^2^ and serving as a natural boundary between Punjab and Baluchistan province. Elevations range from about 100 m to nearly 2,300 m above sea level (a.s.l.). The climate is predominantly arid to semi-arid, with a mean annual rainfall of 100–230 mm, concentrated during the summer monsoon (July–September), and average annual temperatures of 23.5$^\circ$C. The study area spans diverse vegetation zones: arid lowland steppes and Berberis, Acacia and wild Olea species dominate lower to mid slopes, while alpine meadows with hardy grasses and dwarf shrubs (Onobrychis and Acantholimon species) occur above 2000 m a.s.l. Land use is characterized by extensive rangeland grazing – primarily with goats, sheep, cattle, and camels – supplemented by rainfed farming, hill-torrent cultivation, small-scale trade, and remittances. Steep gradients, fragile vegetation, and reliance on rangelands heighten exposure to climate variability, overgrazing, and land-use pressures. Dependence on climate-sensitive resources, infrastructural isolation, and limited scientific documentation underscore the region’s high vulnerability to climate extremes and justify its selection for this study.

### Sampling and data collection

A cross-sectional household survey was conducted between October 2023 and February 2024 using a multi-stage sampling design. At the first stage, the Punjab tribal belt within the districts of Dera Ghazi Khan and Rajanpur was purposively selected, as it constitutes the only tribal region in Punjab Province and encompasses the Koh-e-Suleiman Range. Official voter lists for all seven major tribes inhabiting the region, namely Qaisrani, Buzdar, Khosa, Lund, Lighari, Gorchani, and Mazari,were obtained from the respective district administration offices. In the second stage, three tribes – Buzdar, Lighari, and Gorchani – were randomly selected, and within each, the sample size was proportionally allocated to their respective populations, yielding 107 respondents from Buzdar, 47 from Lighari, and 44 from Gorchani. At the third stage, a snowball sampling approach was employed due to the scattered nature of settlements and seasonal migration patterns, as poor accessibility made it challenging to directly approach respondents. After meetings with village chiefs to explain the study’s purpose and the sample selection criteria (ownership of at least 10 livestock heads and residing in the study area for at least ten years), interviews were conducted with eligible households identified by the village chief. Following the snowball sampling approach, additional respondents were recruited based on referrals from these initial participants within their vicinity or tribal networks. This approach allowed for reaching otherwise inaccessible households, ensuring that a broader and more diverse cross-section of the pastoral population was represented in the sample. A total of 198 household heads were interviewed, proportionally distributed across the three tribes’ total population. Climate perception data were collected using a structured questionnaire that was pilot-tested for clarity, cultural appropriateness, and accuracy of translation into the local Balochi language. Face-to-face interviews, lasting approximately 20–25 min, were conducted in the early mornings or evenings, with the assistance of a local translator to facilitate communication and build trust. The use of snowball sampling may introduce selection bias and limit the statistical representativeness of the sample. Although this approach was necessary due to the lack of name lists for the settlements’ population, the remoteness of scattered settlements, and the seasonal mobility of pastoral households, it may underrepresent households that are less socially connected or more geographically isolated. Consequently, the findings should be interpreted as representative of mobile pastoral and agro-pastoral households in comparable semi-arid mountain contexts, rather than the entire rural population of the region.

### Ethical considerations

All methods were performed in accordance with the relevant guidelines and regulations of the Institutional Biosafety/Bioethics Committee (IBC), University of Agriculture, Faisalabad, Pakistan. Prior to data collection, meetings were held with local village heads and elders to explain the study’s objectives and protocols. These community leaders provided guidance on local customs and helped identify eligible participants according to the sampling framework. Informed, voluntary oral consent was obtained from all respondents before each interview.

### Meteorological data

The Cressman Interpolated High-resolution Gauge-based Gridded Observations (CIHGGO) dataset for Pakistan is a gauge product derived from Pakistan Meteorological Department (PMD) daily station records. CIHGGO is provided on a $0.45^\circ$ grid (approx. 50 km resolution) and distributed in NetCDF format; the original release covers 1980–2018^31^. PMD extended CIHGGO to 1980–2022 using the same Cressman objective analysis as the original release. We received this updated dataset directly from PMD in NetCDF format and processed it via ArcGIS for interpolation. We adopt CIHGGO as our primary precipitation and temperature dataset because it is built directly from national gauges and preserves country-specific spatial structure. To assess robustness, CIHGGO was cross-checked and performed well with ECMWF Reanalysis v5 (ERA5; European Centre for Medium-Range Weather Forecasts) and Modern-Era Retrospective analysis for Research and Applications, Version 2 (MERRA-2; NASA Global Modeling and Assimilation Office), and we referenced the Global Precipitation Climatology Centre (GPCC) as a global observational benchmark^32^.

To align with the study’s focus on regional climatic trends and their societal perception, the CIHGGO data of 10 grid points (see Supplementary Table S1) were aggregated to provide a generalized overview of the study area, rather than analyzing individual grid points or altitude-based variations. This approach was adopted due to the mobile lifestyle of pastoralist communities in the region, with movement patterns and resource use spanning large, heterogeneous landscapes. Accordingly, aggregation was intended to capture regional-scale climatic exposure relevant to the respondents’ livelihoods rather than depict fixed, site-specific conditions. While this approach may smoothen fine-scale, elevation-dependent micro-climatic gradients across the 100–2,300 m a.s.l. range, it was considered more appropriate for representing the climatic conditions experienced across the broader realm of mobile pastoralists.

**Table 1:** Definitions of precipitation and temperature indices (ETCCDI) used in this study (adapted from Zhang et al.^34^).

Index	Code	Definition	Unit
Precipitation			
Total annual precipitation	PRCPTOT	Total annual precipitation of rainy days (RR $\ge$ 1 mm)	mm
Consecutive wet days	CWD	Maximum number of consecutive days with RR $\ge$ 1 mm	Days
Heavy precipitation days	R10 mm	Annual count of days when PRCP $\ge$ 10 mm	Days
Heavy precipitation days	R15 mm	Annual count of days when PRCP $\ge$ 15 mm	Days
Heavy precipitation days	R20 mm	Annual count of days when PRCP $\ge$ 20 mm	Days
Very wet day precipitation	R95p	Annual total precipitation from very wet days (>95th percentile)	mm
Extremely wet day precipitation	R99p	Annual total precipitation from extremely wet days (>99th percentile)	mm
Maximum 1-day precipitation	RX1day	Monthly maximum 1-day precipitation	mm
Maximum 5-day precipitation	RX5day	Monthly maximum 5-day precipitation	mm
Simple daily intensity index	SDII	Mean precipitation amount on wet days (RR $\ge$ 1 mm)	mm
Consecutive dry days	CDD	Maximum number of consecutive days with RR < 1 mm	Days
Temperature			
Warm days	TX90p	Annual count of days when TMAX > 90th percentile	Days
Warm nights	TN90p	Annual count of days when TMIN > 90th percentile	Days
Summer days	SU	Annual count of days when TX > 25$^{\circ }$C	Days
Summer days >30$^{\circ }$C	SU30	Annual count of days when TX > 30$^{\circ }$C	Days
Tropical nights	TR	Annual count when TN > 20$^{\circ }$C	Days
Daily temperature range	DTR	Monthly mean difference between TMAX and TMIN	$^{\circ }$C
Hottest days	TXx	Annual maximum value of TMAX	$^{\circ }$C
Hottest nights	TNx	Annual maximum value of TMIN	$^{\circ }$C
Warm spell duration	WSDI	Annual count of days with $\ge$6 consecutive days when TMAX > 90th percentile	Days
Cold days	TX10p	Annual count of days when TMAX < 10th percentile	Days
Cold nights	TN10p	Annual count of days when TMIN < 10th percentile	Days
Coldest day	TXn	Annual minimum value of TMAX	$^{\circ }$C
Coldest night	TNn	Annual minimum value of TMIN	$^{\circ }$C
Cold spell duration	CSDI	Annual count of days with $\ge$6 consecutive days when TMIN < 10th percentile	Days
Frost days	FD	Annual count of days when TN < 0$^{\circ }$C	Days
Mean maximum temperature	TMAXMEAN	Annual arithmetic mean of daily maximum temperature over the analysis period	$^{\circ }$C
Mean minimum temperature	TMINMEAN	Annual arithmetic mean of daily minimum temperature over the analysis period	$^{\circ }$C
Coldest night of the month	TNN	Monthly minimum value of daily minimum temperature	$^{\circ }$C

#### Meteorological data processing and analysis

Climate data was analyzed using the RClimDex package^33^ implemented in R software (version 4.5.1; R Core Team, 2023). RClimDex is developed by the Expert Team on Climate Change Detection and Indices (ETCCDI) and endorsed by the World Meteorological Organization (WMO) and the World Climate Research Programme (WCRP). The analysis covered 29 extreme climate indices of temperature and precipitation adapted from^34^ and explained in Table 1, covering two periods: a long-term period of 43 years (1980–2022) and the last decade (2013–2022).

To test for monotonic trends in the ETCCDI indices, a nonparametric Mann–Kendall test was performed and the trend magnitude quantified with Sen’s slope. All analyses were conducted in R 4.5.0^35^ using the trend package^36^: mk.test() for Mann–Kendall and sens.slope() for Sen’s slope. For each index, results with $p<0.05$ were classified as “increasing” or “decreasing,” with direction determined by the sign of Kendall’s $\tau$ and the corresponding Sen’s slope.

### Perception–observation accuracy and bias analysis

Seven climate perception indicators were extracted from the household survey, namely temperature, warm spells, cold spells, annual rainfall, rainfall intensity, floods, and drought spells. These were classified as “increase”, “decrease” or “no change”. Observed trends were compared to ETCCDI indices, namely TMAXMEAN, TMINMEAN, TXx, TNx, TX90p and TN90p for temperature; WSDI for warm spells; CSDI for cold spells; PRCPTOT for annual rainfall; SDII for rain intensity; RX1day, RX5day, R10mm, R15mm and R20mm for floods; and CDD for drought spells. Perception and observation where then joined by indicator to create (i) a respondent level accuracy variable (correct vs incorrect) and (ii) a bias variable (overestimate/underestimate/no bias). After that we summarized distributions, tested independence via Pearson $\chi ^2$ (examining standardized residuals), and correlations between each respondent’s accuracy scores (0 = incorrect, 1 = correct) across all seven perception indicators. For data preparation and visualization dplyr (R version 1.1.4) and ggplot2 (R version 3.2.5) packages were used, other analyses were performed using functions of the stats package.

### Drivers of perception accuracy and bias: logistic regression analysis

**Fig. 2:**
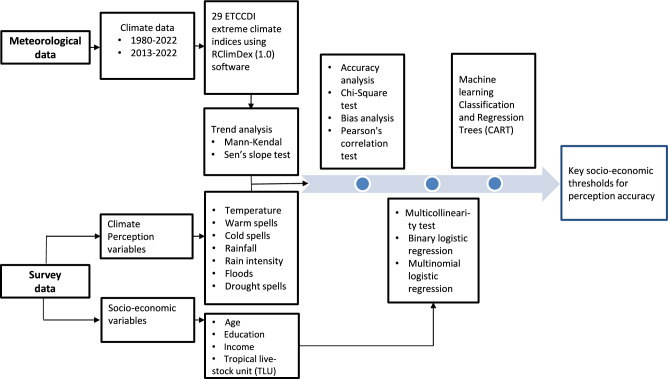
Analytical framework combining meteorological records (ETCCDI indices), household survey data on perceptions and socio-economic variables, and statistical and machine learning models (logistic regression, CART) to identify drivers and thresholds of perception accuracy.

To understand the socio-economic factors that influence climate perceptions, a suite of statistical and machine learning methods was employed. These analyses aimed to model perception accuracy, identify the direction of perceptual bias, and uncover key demographic segments with distinct perception patterns ( Fig. 2).

Before modeling, a multicollinearity check was performed using the Variance Inflation Factor (VIF) to ensure that the socio-economic variables – age, education, income, and herd size  – were not too strongly correlated with each other. This step is crucial for obtaining reliable and interpretable regression estimates; following common guidance, VIF values below 5 were considered acceptable^37^.

Perception accuracy (correct = 1, incorrect = 0) was assessed using a mixed-effects logistic regression. The model included a respondent-level random intercept to account for repeated observations from the same individual, and fixed effects for socio-economic variables. This analysis allowed us to identify which specific characteristics predict the probability of correctly perceiving climate change.

As a complementary analysis, the direction of perceptual bias was examined; bias was treated as an ordinal outcome with three categories: “underestimate”, “no bias”, and “overestimate”. Multinomial logistic regression was used to determine which factors predict a specific type of bias. We tested the proportional-odds assumption and, if violated, utilized a partial-proportional model. This approach allowed to go beyond simple accuracy and understand which population segments tend toward specific types of misperceptions^38^.

#### Classification tree analysis: identifying key variables

To reveal non-linear relationships and interactions that might not be apparent in regression models, Classification and Regression Tree (CART) models were trained for detecting rainfall and drought spells prediction accuracy. CART recursively partitions the data into increasingly homogeneous subgroups based on socio-economic characteristics, identifying the combination of factors that best predict perception accuracy^39^. We used Gini impurity as the splitting criterion and employed 10-fold cross-validation for complexity-parameter (cp) selection; the final tree was pruned at the cp that minimized cross-validated errorusing the rpart package^40^. We report balanced accuracy, confusion matrices, and variable importance (summed Gini reductions across splits) for model interpretability^39^.

All analyses described in Sect. “Perception–observation accuracy and bias analysis” were performed in R 4.5.0 using packages car (multicollinearity), lme4 (binary logistic model), ordinal (multinomial logistic model) and rpart (CART decision trees). The data manipulation and visualization were conducted with the tidyverse, specifically dplyr (version 1.1.4) and ggplot2 (version 3.5.2).

## Results

### Socio-economic profile of the respondents

The socio-economic profile of respondents is summarized in Table 2. The mean age of household heads was 48.4 years with a standard deviation of $\pm 12.5$ years, reflecting a predominantly middle-aged population. Education levels were generally low: 55.6% had no formal schooling, while only 3.5% had completed graduation, highlighting the infrastructural difficulties of the study area. Livestock holdings were a central livelihood asset. On average, households owned $12.4 \pm 9.2$ Tropical Livestock Units (TLU, an animal-equivalent of 250 kg live weight), with $8.0 \pm 6.5$ TLU in small ruminants (sheep, goats) and $4.4 \pm 4.5$ TLU in large ruminants (cattle, camels). Livestock also contributed substantially to household income, with a mean livestock-derived income of USD 1,767 $\pm 2,554$. The mean total annual household income was USD 3,461 $\pm 3,434$, underscoring both the economic reliance on livestock and the high variability in livelihood resources among pastoral households.

**Table 2:** Socio-economic characteristics of the surveyed households (n = 198) in the Koh-e-Suleiman Range, Pakistan.

Variables	Category / unit	N (%)	Mean	SD
Age (years)	Continuous	198 (100)	48.4	12.5
Education (years of schooling)	0 = No schooling	110 (55.6)	–	–
	1 = Primary	52 (26.3)	–	–
	2 = Secondary	29 (14.6)	–	–
	3 = Graduation	7 (3.5)	–	–
TLU-SR	Continuous	198 (100)	8.0	6.5
TLU-LR	Continuous	198 (100)	4.4	4.5
TLU-Total	Continuous	198 (100)	12.4	9.2
Income livestock (USD)	Continuous	198 (100)	1767	2554
Total income (USD)	Continuous	198 (100)	3461	3434

Note: TLU-SR = Tropical Livestock Units for small ruminants (goats, sheep); TLU-LR = Tropical Livestock Units for large ruminants (cattle, camels); TLU-Total = combined livestock holdings converted to TLU equivalents. Income values were converted to US dollars (USD) at the prevailing exchange rate during the survey period

### Long term (1980–2022) and short term (2013–2022) climate trends

To analyze long-term climate variability and recent trends, we examined climate data over two distinct periods: a long-term period from 1980–2022 and a more recent, short-term period from 2013–2022. The results of this analysis are summarized in Table 3. The 1980–2022 analysis provides a broad historical context, whereas the 2013–2022 analysis captures more recent climate variability and potential intensification, with particularly pronounced changes in temperature-related indices such as warm spells and maximum temperature extremes. Accordingly, results from the 2013–2022 period are interpreted as reflecting recent variability and possible intensification rather than definitive long-term trends, as statistical inference is limited by the shorter record length and high interannual variability.

**Table 3:** Mann–Kendall trend test and Sen’s slope estimates for precipitation and temperature indices over 1980-2022 and 2013-2022.

Index	1980-2022			2013-2022		
	$\tau$	*p*-value	Sen’s slope	$\tau$	*p*-value	Sen’s slope
Precipitation						
PRCPTOT	0.262	0.013*	4.30	0.156	0.592	16.85
CWD	0.105	0.347	0	0.025	1.000	0
R10mm	0.325	0.002**	0.190	0.200	0.474	0.714
R15mm	0.228	0.039*	0.080	0.263	0.356	0.500
R20mm	0.131	0.249	0	−0.070	0.855	0
R95p	0.103	0.340	0.653	−0.067	0.858	-3.525
R99p	0.105	0.388	0	0.218	0.476	0
RX1day	0.073	0.496	0.093	−0.022	1.000	-0.150
RX5day	0.147	0.167	0.345	0.067	0.858	1.867
SDII	0.218	0.042*	0.041	0.000	1.000	0
CDD	0.134	0.209	0.567	−0.022	1.000	−1.000
Temperature						
TX90p	0.116	0.276	0.292	0.556	0.032*	3.670
TN90p	0.214	0.044*	0.530	0.644	0.012*	7.230
SU	0.173	0.107	0.308	0.022	1.000	0.667
SU30	0.215	0.045*	0.389	0.556	0.032*	2.500
TR	0.065	0.550	0.083	0.414	0.123	2.333
DTR	-0.065	0.537	-0.005	−0.539	0.039*	-0.059
TXx	-0.159	0.139	-0.018	0.092	0.785	0.022
TNx	-0.077	0.483	-0.007	0.000	1.000	0
WSDI	0.292	0.009**	0.200	0.768	0.003**	3.143
TX10p	-0.032	0.769	-0.081	0.111	0.720	0.762
TN10p	-0.192	0.071	-0.443	−0.378	0.152	−3.419
TXn	0.031	0.777	0.006	−0.046	0.927	0
TNN	0.161	0.134	0.029	0.422	0.107	0.350
CSDI	-0.200	0.083	0	−0.669	0.019*	−0.857
FD	-0.076	0.535	0	−0.542	0.069	0
TMAXMEAN	0.092	0.391	0.007	0.244	0.371	0.084
TMINMEAN	0.208	0.050*	0.015	0.644	0.012*	0.186

$\tau$ = Kendall’s tau (direction/strength of monotonic trend; + increasing, – decreasing); *p* = significance (* $p{\text{ }} < 0.05$, ** $p{\text{ }} < 0.01$); Sen’s slope = annual rate of change (units per year)

#### Temperature

In the long-term (1980-2022) data set, warm-extreme indices such as the warm spell duration index (WSDI: $\tau = 0.292, p = 0.009$), annual count of summer days above $30^{\circ }$C (SU: $\tau = 0.215, p = 0.045$), frequency of unusual warm nights (TN90p: $\tau = 0.214, p = 0.044$), and mean minimum temperature (typically night-time) over the analysis period (in $^\circ$C) (TMINMEAN: $\tau = 0.208, p = 0.050$) increased over the long term. Daytime peak intensity indices were not significant.

In the recent decade, warming intensified and became more asymmetric, with frequency of unusual warm days (TX90p: $\tau = 0.556, p = 0.032$), frequency of unusual warm nights (TN90p: $\tau = 0.644, p = 0.012$), warm spell duration index (WSDI: $\tau = 0.768, p = 0.003$), annual count of summer days above $30^{\circ }$C (SU30: $\tau = 0.556, p = 0.032$), and mean minimum temperature (TMINMEAN: $\tau = 0.644, p = 0.012$) all increased. Daily temperature range (DTR: $\tau = -0.539, p = 0.039$) and cold spell duration (CSDI: $\tau = -0.669, p = 0.019$) decreased, indicating fewer cold spells and a narrower day–night temperature range driven by rising nighttime minima. TMAXMEAN remained non-significant. Taken together, short-term signals showed more hot days (TX90p) and warmer nights (TMINMEAN), that is a higher frequency and duration of heat exposure, with intensity increases concentrated at night.

#### Precipitation

Over the period 1980–2022, total annual precipitation (PRCPTOT: $\tau = 0.262$, $p = 0.013$; Sen = +4.30 mm$\cdot$yr$^{-1}$) showed a significant increase. Rain intensity indices, annual counts of days with precipitation <10 mm (R10mm: $\tau = 0.325$, $p = 0.002$), annual counts of days with precipitation <15 mm (R15mm: $\tau = 0.228$, $p = 0.039$), and the simple daily intensity index (SDII: $\tau = 0.218$, $p = 0.042$) all increased significantly, while indices for extremely wet periods such as very wet days (R95p; annual total precipitation of days exceeding the 95th-percentile daily rainfall threshold, mm), extremely wet days (R99p; annual total precipitation of days exceeding the 99th-percentile daily rainfall threshold, mm), maximum 1-day precipitation (RX1day; largest single-day precipitation amount in a year, mm), and maximum 5-day precipitation (RX5day; largest total precipitation accumulated over any consecutive 5-day period in a year, mm) were not significant. The consecutive dry days (CDD) indicator also showed nonsignificant results.

In the period 2013–2022, no precipitation index remained statistically significant (all $\textit{p}>0.05$). For PRCPTOT specifically, the trend was not significant ($\tau =0.156,\ p=0.592$) despite a larger estimated Sen slope ($16.9$ units yr$^{-1}$), indicating a positive direction that did not reach significance, likely due to the short window and high year-to-year rainfall variability. Directionally, moderate-to-heavy event metrics (R10mm, R15mm, RX5day) showed positive $\tau$, whereas the very-wet/exreme-wet day indicator RX1 remained flat to slightly negative. CDD was slightly negative and consecutive wet days (CWD)remained unchanged.

The Mann–Kendall $\tau$ heatmap (see Supplementary Fig. S1) reflects the intensification patterns: brighter color saturation in the last-decade column indicates larger absolute $\tau$ values. Temperature rises clearly appear in the period 2013-2022: more hot days and nights, longer warm spells, and shrinking day–night ranges indicate heightened heat exposure led by rising night-time minima. In contrast, precipitation related indices in the period 2013–2022 showed mixed, non-significant signals, suggesting attenuation of earlier wetting and elevated decadal variability.

### Perception accuracy and bias as determined by comparison with meteorological data

Household perception data was compared with the long-term meteorological record (1980–2022) for subsequent analysis. This alignment enables respondent-level accuracy and bias assessment against objectively observed climate trends. The survey participants showed widespread recognition of climate change: for temperature, 83.3% of respondents perceived an increase, and 82.8% noted more frequent warm spells. Cold spells were reported to have decreased by 71.7% of the interviewees. Rainfall perceptions were more mixed: 60.6% reported increased rainfall, and 84.8% observed greater rain intensity. A strong majority of 87.9% perceived more frequent floods. However, only 24.7% accurately identified trends of drought spells, with most overestimating their frequency.

Agreement with observed trends (correct) was highest for floods (87.9%), rain intensity (84.8%), temperature chnages (83.3%), and warm spells (82.8%); moderate for cold spells (71.7%) and rainfall (60.6%); and lowest for drought spells, where only 24.7% of perceptions were correct. The bias profile shows a systematic overestimation of drought spells (60.1%), and a systematic underestimation of rainfall (27.3%); other variables were mostly “accurate” with small shares of “misjudged” observations (Table 4).

**Table 4:** Distribution of respondents’ perception response, accuracy, and bias level across seven climate variables (n = 198) compared to 1980–2022 meteorological data in the Koh-e-Suleiman Range, Pakistan.

Variable	Perception response (%)			Accuracy level (%)		Bias level (%)		
	Increase	Decrease	No change	Correct	Incorrect	Misjudged	Overestimate	Underestimate
Temperature-related indicators								
Temperature	83.3	1.0	15.7	83.3	16.7	15.7	0.0	1.0
Warm spells	82.8	1.0	16.2	82.8	17.2	16.2	0.0	1.0
Cold spells	8.6	71.7	19.7	71.7	28.3	19.7	8.6	0.0
Precipitation-related indicators								
Rainfall	60.6	27.3	12.1	60.6	39.4	12.1	0.0	27.3
Rain intensity	84.8	2.0	13.1	84.8	15.2	13.1	0.0	2.0
Floods	87.9	0.0	12.1	87.9	12.1	12.1	0.0	0.0
Drought-related indicators								
Drought spells	60.1	15.2	24.7	24.7	75.3	0.0	60.1	15.2

“Misjudged” indicates responses of “no change” when the observed trend was increasing or decreasing; “overestimate” and “underestimate” indicate directional disagreement with observed trends.

Results of the Chi-square test confirmed strong associations between variable categories and the perceptions, which differed systematically across variables ($\chi ^{2} = 655.93$, df = 12, $p < 0.001$). Standardized residuals highlighted the clearest agreements and mis-matches: a large positive residual was obtained for “cold-spell decrease” (+19.00), indicating many more ‘decrease’ responses than expected under the assumption of independence (if responses were independent of the climate variable). Positive residuals for “floods increase” (+3.57) and “rain-intensity increase” (+3.32) indicated strong alignment of perceptions with observed trends. Respondents significantly underestimated the likelihood of “temperature decrease” and “warm-spell decrease” (both with residuals of $-5.39$), which aligns with the broader trend of global warming. In contrast, responses related to drought spells showed only minor deviations from expectations — the largest being a slight over-selection of “no change” ($+2.95$), indicating mixed perceptions among participants (see Supplementary Fig. S2).

Perception accuracy for temperature and warm spells were strongly correlated ($r = 0.52, p < 0.001$), while accuracy of the perception of drought spells was inversely related to those of the occurrence of floods ($r = -0.47, p < 0.001$), rain intensity ($r = -0.35, p < 0.001$), and rainfall ($r = -0.23, p < 0.001$), suggesting perceived trade-offs or event-salience effects (see Supplementary Fig. S3).

### Perception–observation alignment

The comparison of perception indicators with ETCCDI indices reveals a broad alignment between local perceptions and observed climatic trends during 1980–2022 (Table 5). Perceived increases in temperature and warm spells were consistent with significant warming signals across indices such as mean minimum temperature (TMINMEAN), warm nights (TN90p), annual count of summer days above $30^{\circ }$C (SU30), and warm spell duration index (WSDI). Similarly, the majority of respondents accurately perceived declines in cold spells, matching the negative trend in the cold spells duration index (CSDI). Rainfall-related perceptions also aligned well: both total precipitation (PRCPTOT) and rainfall intensity (SDII) showed long-term increases consistent with the respondents’ views. Perception of floods was also largely correct, as increases in heavy-rainfall indices (SDII, R10mm, R15mm) supported the observations. In contrast, drought spells perception showed the greatest mismatch: while the drought spells index (CDD) indicated no significant change, 60% of the respondents perceived an increase of drought spells. This divergence highlights an overestimation bias toward the frequency of drought spells, in contrast to the strong agreement observed for temperature, rainfall, and flood-related variables.

**Table 5:** Correspondence between ETCCDI indices and climate perceptions of pastoralists (n = 198) in the Koh-e-Suleiman Range, Pakistan.

Perception variable	ETCCDI index	(1980–2022) Trend	Objective direction	Perceived direction	Perception match
Temperature	TMAXMEAN	0.391	Increase	Increase	Match
	TMINMEAN	0.050*			
	TN90P	0.044*			
	SU30	0.045*			
	DTR	0.537			
Warm spells	WSDI	0.009**	Increase	Increase	Match
Cold spells	CSDI	0.083	Decrease	Decrease	Match
Rainfall	PRCPTOT	0.013*	Increase	Increase	Match
Rain intensity	SDII	0.042*	Increase	Increase	Match
Floods	R10mm	0.002**	Increase	Increase	Match
	R15mm	0.039*			
Drought spells	CDD	0.209	No change	Increase	Mismatch

Objective direction = meteorological trend; perceived direction = household survey response. * $p < 0.05$, ** $p < 0.01$. “Match” indicates alignment between observed and perceived directions; “Mismatch” indicates divergence.

### Determinants of perceptional accuracy and bias

Binary logistic regression revealed a strong link between certain socio-economic variables and the accuracy of perceiving specific climate variables. Before proceeding to the regression analysis, multicollinearity checks indicated low correlations among variables ($r < 0.5$) and acceptable VIF values (1.02–1.25). The analysis (Table 6) showed significant positive association between a greater number of animals (in terms of TLU) and the owner accurately perceiving rising temperatures (OR $=$ 1.070, $p = 0.035$). For cold spells, accuracy was associated with age (OR $=$ 1.032, $p = 0.027$), education (OR $=$ 1.129, $p = 0.009$), and income ($p = 0.017$), indicating that older, more educated, and better-off individuals perceived these events more accurately. Yet no other socio-economic variable significantly predicted perception of rainfall, flood, or drought spells, although education showed a marginal significant association with rain intensity ($p = 0.068$).

To gain a more granular understanding of the specific type of perception error (overestimation or underestimation), a multinomial logistic regression was employed, which caters for dependent variables with more than two categories. Regarding temperature, the results revealed that higher education levels significantly reduced the underestimation of warming (OR $=$ 0.16, $p < 0.001$), while age (OR $=$ 0.97, $p < 0.001$), education (OR $=$ 0.95, $p < 0.001$), and total TLU (OR $=$ 0.93, $p < 0.001$) were associated with higher overall temperature accuracy. For rainfall, the odds of misjudgment increased slightly with age (OR $=$ 1.03, $p < 0.001$) but decreased significantly with education (OR $=$ 0.85, $p < 0.001$). Furthermore, higher education was associated with an overestimation of rain intensity (OR $=$ 1.08, $p < 0.001$), and greater livestock holdings (TLU) were associated with a slight increase in flood overestimation (OR $=$ 1.02, $p < 0.001$). Similarly, higher income was associated with an increased likelihood of overestimating cold spells (OR $\approx$ 1.00, $p < 0.001$). Collectively, these findings indicate that education is associated with higher climate perception accuracy by reducing misjudgments, whereas greater reliance on pastoral activities, indicated by higher animal numbers (TLU), is associated with overestimation of precipitation-related phenomena. These associations should be interpreted as correlational rather than causal, reflecting the observational nature of the analysis.

**Table 6:** Significant predictors of perception accuracy and bias from binary (BLR) and multinomial (MLR) logistic regression models.

Dependent variable	Model	Outcome category	Predictor(s)	Odds ratio (95% CI)	*p*-value	Interpretation
Temperature	BLR	Accuracy	TLU	1.070 (not reported)	0.035	Larger herds associated with higher odds of correctly perceiving warming.
	MLR	Misjudgment	Age	0.97 (0.96–0.98)	$<0.001$	Older respondents less likely to misjudge.
			Education	0.95 (0.93–0.97)	$<0.001$	Higher education reduces misjudgment.
			TLU	0.93 (0.91–0.95)	$<0.001$	Livestock wealth decreases misjudgment odds.
		Underestimation	Education	0.16 (0.14–0.18)	$<0.001$	Education strongly protects against underestimation.
Cold spells	BLR	Accuracy	Age	1.032 (not reported)	0.027	Older respondents are more likely to correctly perceive cold spells.
			Education	1.129 (not reported)	0.009	Higher education improves cold-spell accuracy.
			Income	–	0.017	Higher income improves cold-spell accuracy.
	MLR	Overestimation	Income	1.00 (1.0000–1.000002)	$<0.001$	Marginal effect: wealth linked to slight overestimation.
Rainfall	BLR	Accuracy	–	–	$>0.10$	No significant predictors found.
	MLR	Misjudgment	Age	1.03 (1.01–1.05)	$<0.001$	Older respondents have slightly higher odds of misjudging rainfall.
			Education	0.85 (0.82–0.88)	$<0.001$	Higher education reduces rainfall misjudgment.
Rain intensity	BLR	Accuracy (marginal)	Education	–	0.068	A marginal negative association between education and accuracy.
	MLR	Overestimation	Education	1.08 (1.05–1.11)	$<0.001$	More education predicts overestimation.
Floods	BLR	Accuracy (marginal)	Age	–	0.063	A marginal positive association between age and accuracy.
	MLR	Overestimation	TLU	1.02 (1.01–1.03)	$<0.001$	Larger herds linked to a slight increase in flood overestimation.
Warm spells	BLR	Accuracy	–	–	$>0.10$	No significant predictors found.
Drought spells	BLR	Accuracy	–	–	$>0.10$	No significant predictors found.

BLR, Binary logistic regression; MLR, Multinomial logistic regression. Odds ratios (OR) with 95% confidence intervals show how predictors (age, education, income, TLU) affect perception accuracy or bias. OR > 1 means the predictor increases the likelihood of correct perception (or a given bias), while OR < 1 means it reduces the likelihood. Only statistically significant results ($p < 0.05$) are reported. The reference category for all logistic regression models is “Correct perception.” For multinomial logistic regression models, “Correct perception” was used as the reference outcome category; all odds ratios are interpreted relative to this baseline. Continuous predictors (age, income, TLU) were entered as continuous variables.

### Classification and regression tree analysis

Following the regression models, a Classification and Regression Tree (CART) analysis was performed to examine the hierarchical and non-linear effects of socio-economic predictors on perception accuracy. Whereas logistic regressions identified significant independent associations, the non-parametric machine learning method CART revealed specific thresholds at which predictors such as income, TLU, and age shape misperception. Misperceptions were particularly pronounced for drought spells, whereas rainfall was comparatively less misperceived by respondents.

#### Rainfall

The CART model highlighted total TLU as the most influential predictor for rainfall perceptions. Respondents with fewer than 20 TLU were substantially less likely to perceive rainfall trends accurately. Among those with $\ge 20$ TLU, perception accuracy exhibited age-related variation: individuals aged 36–60 years exhibited the highest accuracy, while those aged $< 36$ also performed relatively well. In contrast, respondents $> 60$ years displayed more inconsistent accuracy, suggesting that both herd size and environmental experience tend to increase and improve, respectively, with age. However, perception accuracy appeared to decline beyond a threshold of 60 years (see Supplementary Fig. S4).

#### Drought spells

For drought spells, income emerged as the primary splitting variable. Respondents earning less than USD $2,759 annually showed the lowest prediction accuracy, with over 75% misperceiving the frequency of drought spells. Again, age compounded this effect: within the low-income group, respondents younger than 51 years were especially prone to overestimating the occurrence of drought spells, while older individuals’ perceptions showed slightly better alignment with observed records (see Supplementary Fig. S5).

## Discussion

The Koh-e-Suleiman Range, as part of the wider Hindu Kush Himalayan (HKH) system, mirrors the accelerated climate changes reported across Asian mountain regions. Similarly to the findings of the Himalayas and the Hindu Kush, our results indicate strengthening of warming signals and shifting precipitation patterns. Over the last decade, warming has amplified, with increases in warm extremes and declines in cold spells This is consistent with asymmetric warming across South Asia’s highlandsdue to stronger night-time than daytime temperature increases^41,42^. Changes in precipitation patterns, however, are more complex. Evidence from Pakistan’s northern highlands shows declining totals and decreasing frequency of heavy rainfall events (e.g., Rx1day, R95p, R99p, and SDII) over the last three decades^43^.

Our two-period analysis (1980–2022 vs. 2013–2022) captures both the long-term context and recent accelerations, highlighting how even modest changes in rainfall intensity can have major hydrological impacts in semi-arid mountains prone to flash floods and droughts^44^. While the 1980–2022 period provides a robust basis for identifying long-term climatic trends, the shorter 2013–2022 period reflects recent variability and potential intensification rather than definitive trend signals, given its limited temporal length. Analyzing long and short-term patterns in parallel helped to explain why drought spells were often misperceived, while rainfall intensity was assessed more accurately by respondents.

Comparable patterns are evident in Nepal, Bhutan, and northern India, where long-term precipitation intensification has been accompanied by increasing variability in recent decades^45–47^. In Bhutan, ETCCDI analysis revealed heterogeneous precipitation trends, with some districts experiencing more frequent dry spells while others recorded intensifying extreme rainfall, underscoring the localized risks for agriculture and hydropower-dependent economies^46^. Similarly, nationwide assessments in Nepal identified marked east–west contrasts in precipitation extremes: pre-monsoonal rainfall and high-intensity events have increased in the western mountains and hills, while central and eastern regions experienced declining trends alongside prolonged dry spells^45^. These findings resonate with our results from the Koh-e-Suleiman Range, where modest but significant shifts in rainfall intensity and variability have major hydrological impacts in semi-arid mountain contexts^44^. Altogether, the evidence affirms that Pakistan’s semi-arid mountains are embedded in a wider regional climatic mosaic characterized by intensification, asymmetry, and growing unpredictability.

Pastoral and agro-pastoral households in the Koh-e-Suleiman Range perceived rises in temperature, warm spells, and flood frequency with relatively high accuracy. This pattern is consistent with findings from other South Asian mountain regions, where visible and frequent hazards are more readily recognized because of their immediate livelihood impacts, such as livestock heat stress, grazing land degradation, and flood damage to homes and infrastructure^48^. At the same time, the geographic context shapes how other signals are interpreted. Hamilton and Keim^49^ demonstrated strong regional variation in climate change perceptions in the United States, a finding that resonates here: the semi-arid mountain setting of the Koh-e-Suleiman appears to heighten misperceptions of drought spells and rainfall compared to more conspicuous climatic events such as warm spells and floods.

In contrast, perceptions of changes in rainfall and drought spells were more ambiguous. Many respondents reported both increasing rainfall and increasing drought spells, a paradox often reported as increased drought alongside more intense rainfall in Bangladesh^48^, increasing rainfall and longer dry spells in Nepal^45^, and erratic/variable rainfall with increased droughts in Ethiopia^50^. The reason lies in how people experience climate: short wet or dry spells weigh more heavily in memory than gradual long-term averages. The drought spells index (CDD) did not show statistically significant trends, yet short episodes of drought spells, for example in 2021–2022, left strong impressions despite the absence of a long-term drying pattern. Accordingly, drought spells are included here to evaluate perception–observation mismatch rather than to infer a statistically significant long-term drying trend, as short, high-impact events emphasized in drought spells assessment and prediction studies can disproportionately shape lived experience and risk perception. This suggests that experiential and socio-cultural factors, combined with psychological perception biases, account for more variance in risk perception than cognitive evaluations alone, as extreme short-term shocks tend to anchor memories more strongly than statistical norms^51^.

The misperception of drought spells thus reflects not a failure of correct perception, but an experiential interpretation of climate reality. As indicated by the results of the CART analysis, low-income and younger respondents equated any dry spell with drought spells because it translates directly into fodder shortages for their animals and thus reduced livestock-derived income.

Education, age, income, and herd size emerged as the main drivers of perception accuracy and bias. Education consistently improved accuracy, particularly by reducing misjudgments about cold spells and rainfall. Similar patterns have been reported from the South Punjab region in Pakistan, where age, education, farming experience, and off-farm income significantly shaped risk attitudes and perceptions^52^. A study from Bangladesh likewise identified farm size, income, education, and received training as important factors associated with climate perception^48^. Together, these findings confirm that education and knowledge access are central to consolidating the interpretation of variable climatic signals and foster adaptive capacity.

Age and livelihood resources also influenced perceptions, though not linearly. Older respondents were less prone to some misjudgments, but CART results suggest that younger and middle-aged groups sometimes perceived rainfall more accurately than the oldest cohort, which may reflect generational differences in attention, memory, or reliance on ecological cues^17,53^.

Methodologically, combining regression models with classification approaches such as CART has proven valuable in advancing this discussion. Regression analyses capture the broad influence of socio-economic factors on perception accuracy, while machine learning models identify threshold effects and interactions that are less visible in linear models. Together, these approaches move beyond descriptive accounts to highlight specific groups most at risk of misinterpreting rainfall and signals of drought spells.

Recognizing these dynamics has important policy implications. Interventions that treat local perceptions as “misunderstandings” risk overlooking their roots in vulnerability and livelihood dependence. Instead, targeted communication strategies, participatory climate services, and early warning systems tailored to resource-poor households can help bridge the gap between empirical climate information and experiential knowledge^54–56^. Such strategies are increasingly emphasized in climate adaptation planning, as they ensure that technical data resonate with the realities of those most exposed to climatic risk^57^.

## Conclusion

This study provides systematic assessments of climate change perceptions against meteorological evidence in the Koh-e-Suleiman Range, a fragile and understudied mountain ecosystem of Pakistan. Long-term climate data analysis revealed intensifying warming signals, particularly asymmetric night-time warming, alongside increasingly variable rainfall. While pastoral and agro-pastoral households accurately perceived several highly recognizable climate hazards such as rising temperatures, warm spells, floods, and heavy rainfall, misperception was common for drought spells and rainfall variability. These mismatches reflect the influence of short-term anomalies, experiential memory, and livelihood conditions.

The integration of regression and CART analysis helped to understand how perception accuracy is associated with socio-economic variables, most notably herd size, income, and age. The combined use of statistical and machine-learning methods therefore provides a robust and adaptable framework for reconciling perception and observation data in order to identify non-linear relationships that may be overlooked by conventional approaches alone.

These findings demonstrate that perception–observation mismatches are structured, systematic, and closely linked to lived exposure, livelihood vulnerability, and recently experienced climate extremes rather than due to random error or lack of awareness. By explicitly identifying which extremes are accurately perceived and which are not, and how socio-economic realities shape these patterns, this study provides an empirical basis for interpreting local climate perceptions as meaningful indicators of vulnerability and risk in semi-arid mountain systems.

## Supplementary Information

Supplementary Information.

## Data Availability

The data is available upon request from the corresponding author for scientific purposes only.

## References

[1] Kohler, T. & Maselli, D. *Mountains and Climate Change: From Understanding to Action* (University of Bern, Centre for Development and Environment, 2009).

[2] IPCC. Summary for policymakers. In Lee, H. & Romero, J. Climate change 2023: synthesis report. In *Contribution of Working Groups I, II and III to the Sixth Assessment Report of the Intergovernmental Panel on Climate Change*, 1–34 (IPCC, 2023). 10.59327/IPCC/AR6-9789291691647.001

[3] United Nations Development Programme. *The Next Frontier: Human Development and the Anthropocene* (United Nations Development Programme, 2020).

[4] Perkins, K. M. et al. International perspectives on the pedagogy of climate change. Journal of Cleaner Production 200, 1043–1052 (2018). 10.1016/j.jclepro.2018.07.296

[5] Adil, L., Eckstein, D., Künzel, V. & Schäfer, L. Climate Risk Index 2025: Who suffers most from extreme weather events? Climate Risk Index 2025 Report, 74 (2025). Accessed 26 January 2026.

[6] Asare-Nuamah, P. & Botchway, E. Comparing smallholder farmers’ climate change perception with climate data: The case of Adansi North District of Ghana. *Heliyon* **5**, e03065. 10.1016/j.heliyon.2019.e03065 (2019). 31890976 10.1016/j.heliyon.2019.e03065 PMC6928297

[7] Deressa, T. T., Hassan, R. M. & Ringler, C. Perception of and adaptation to climate change by farmers in the Nile Basin of Ethiopia. *J. Agric. Sci.* **149**, 23–31. 10.1017/S0021859610000687 (2011).

[8] Gandure, S., Walker, S. & Botha, J. J. Farmers’ perceptions of adaptation to climate change and water stress in a South African rural community. *Environ. Develop.* **5**, 39–53. 10.1016/j.envdev.2012.11.004 (2013).

[9] Alam, G. M. M., Alam, K. & Mushtaq, S. Climate change perceptions and local adaptation strategies of hazard-prone rural households in Bangladesh. *Clim. Risk Manag.* **17**, 52–63. 10.1016/j.crm.2017.06.006 (2017).

[10] Grothmann, T. & Patt, A. Adaptive capacity and human cognition: The process of individual adaptation to climate change. *Global Environ. Change* **15**, 199–213. 10.1016/j.gloenvcha.2005.01.002 (2005).

[11] Tambo, J. A. & Abdoulaye, T. Smallholder farmers’ perceptions of and adaptations to climate change in the Nigerian Savanna. *Region. Environ. Change* **13**, 375–388. 10.1007/s10113-012-0351-0 (2013).

[12] Fernández-Giménez, M. E., Batkhishig, B., Batbuyan, B. & Ulambayar, T. Lessons from the Dzud: Community-based rangeland management increases the adaptive capacity of Mongolian herders to winter disasters. *World Develop.* **68**, 48–65. 10.1016/j.worlddev.2014.11.015 (2015).

[13] Opiyo, F. et al. Determinants of perceptions of climate change and adaptation among Turkana pastoralists in northwestern Kenya. *Clim. Develop.* **8**, 179–189. 10.1080/17565529.2015.1034231 (2016).

[14] Howe, P. D., Mildenberger, M., Marlon, J. R. & Leiserowitz, A. Geographic variation in opinions on climate change at state and local scales in the USA. *Nat. Clim. Change* **5**, 596–603. 10.1038/nclimate2583 (2015).

[15] Haq, S. M. A. & Ahmed, K. J. Is fertility preference related to perception of the risk of child mortality, changes in landholding, and type of family? A comparative study on populations vulnerable and not vulnerable to extreme weather events in Bangladesh. *Popul. Res. Policy Rev.* **58**, 1–23. 10.1353/prv.2019.0007 (2019).

[16] Hasan, M. K. & Kumar, L. Comparison between meteorological data and farmer perceptions of climate change and vulnerability in relation to adaptation. *J. Environ. Manag.* **237**, 54–62. 10.1016/j.jenvman.2019.02.028 (2019). 10.1016/j.jenvman.2019.02.028 30780055

[17] Poortinga, W., Whitmarsh, L., Steg, L., Böhm, G. & Fisher, S. Climate change perceptions and their individual-level determinants: A cross-European analysis. *Global Environ. Change* **55**, 25–35. 10.1016/j.gloenvcha.2019.01.007 (2019).

[18] Manandhar, S., Pandey, V. P. & Kazama, F. Hydro-climatic trends and people’s perceptions: Case of Kali Gandaki River Basin. *Nepal. Clim. Res.* **54**, 167–179. 10.3354/cr01108 (2012).

[19] Meldrum, G. et al. Climate change and crop diversity: Farmers’ perceptions and adaptation on the Bolivian Altiplano. *Environ. Develop. Sustain.* **20**, 703–730. 10.1007/s10668-016-9906-4 (2018).

[20] Fahad, S. & Wang, J. Farmers’ risk perception, vulnerability, and adaptation to climate change in rural Pakistan. *Land Use Policy* **79**, 301–309. 10.1016/j.landusepol.2018.08.018 (2018).

[21] Ullah, H., Rashid, A., Liu, G. & Hussain, M. Perceptions of mountainous people on climate change, livelihood practices and climatic shocks: A case study of swat district. *Pakistan. Urban Clim.* **26**, 244–257. 10.1016/j.uclim.2018.10.003 (2018).

[22] Mehmood, M. S. et al. An evaluation of farmers’ perception, awareness, and adaptation towards climate change: A study from Punjab province. *Pakistan Ciência Rural* **52**, e20201109. 10.1590/0103-8478cr20201109 (2022).

[23] Shah, S. A. A., Mehmood, M. S., Muhammad, I., Ahamad, M. I. & Wu, H. Adapting harvests: A comprehensive study of farmers’ perceptions, adaptation strategies, and climatic trends in Dera ghazi khan. *Pakistan. Sustain.* **16**, 7070. 10.3390/su16167070 (2024).

[24] Mobeen, M., Kabir, K. H., Schneider, U. A., Ahmed, T. & Scheffran, J. *Climate Change Perception, Adaptation, and Constraints in Irrigated Agriculture in Punjab and Sindh, Pakistan*. Mitigation and Adaptation Strategies for Global Change 30, 23 (2025). 10.1007/s11027-025-10212-1. Open access (CC BY 4.0).

[25] Abid, M., Schneider, U. A. & Scheffran, J. Farmer perceptions of climate change, observed trends and adaptation of agriculture in Pakistan. *Environ. Manage.* **63**, 110–123. 10.1007/s00267-018-1113-7 (2019). 30341722 10.1007/s00267-018-1113-7

[26] Ahmad, B., Shah, S. A. A. & Khan, N. A. People’s perception of climate change impacts in the upper Indus Basin, Pakistan. *Climate* **12**, 73. 10.3390/cli12050073 (2024).

[27] Khan, W., Jamal, M. H. B. et al. *Projected Bioclimatic Shifts in Pakistan: A cmip6 Ensemble Analysis Under Shared Socioeconomic Pathways*. Climate Dynamics. CMIP6-based projection study. (2024).

[28] Khan, N. et al. A novel index for assessing the compound drought, heat waves, and air pollution extremes. *Urban Clim.* **65**, 102726. 10.1016/j.uclim.2025.102726 (2026).

[29] Khan, N., Kamruzzaman, M. & Shahid, S. Diurnal pattern of heat stress over South Asia: A wet bulb globe temperature-based analysis from 1984 to 2023. International Journal of Climatology. Regional heat stress diagnostic study. (2025).

[30] Khan, N., Shahid, S., Sharafati, A. et al. *Determination of cotton and wheat yield using the standard precipitation evaporation index in Pakistan*. Theoretical and Applied Climatology. Crop-yield response to drought indicators. (2021).

[31] Ahmad, B., Bukhari, S. A. A. & Cheema, S. B. *Generation of Cressman Interpolated High-Resolution Gauge-Based Gridded Observations (Cihggo) for Climatic Variables Using in-situ Data Over Pakistan*. Technical Report, Pakistan Meteorological Department, (2020). Accessed 2025–09-01.

[32] Iqbal, S. W. et al. Performance evaluation and comparison of observed and reanalysis gridded precipitation datasets over Pakistan. *Theoret. Appl. Climatol.* **149**, 1093–1116. 10.1007/s00704-022-04100-w (2022).

[33] Zhang, X. & Yang, F. RClimDex (1.0) User Manual. Climate Research Branch, Environment Canada, Downsview, Ontario, (2004). Accessed via ACMAD RCC: https://rcc.acmad.org/procedure/RClimDexUserManual.pdf

[34] Zhang, X. et al. Indices for monitoring changes in extremes based on daily temperature and precipitation data. *Wiley Interdiscip. Rev. Clim. Change* **2**, 851–870. 10.1002/wcc.147 (2011).

[35] R Core Team. R: *A Language and Environment for Statistical Computing*\ (R Foundation for Statistical Computing, 2024).

[36] Pohlert, T. *Trend: Non-Parametric Trend Tests and Change-Point Detection* (R package, 2020).

[37] Hair, J. F., Black, W. C., Babin, B. J. & Anderson, R. E. *Multivariate Data Analysis* 8 edn. (Cengage, 2019).

[38] Menard, S. W. Applied logistic regression analysis. In *Quantitative Applications in the Social Sciences*, Vol. 106 2nd edn. (SAGE Publications, 2002).

[39] Breiman, L., Friedman, J. H., Olshen, R. A. & Stone, C. J. *Classification and Regression Trees* (Wadsworth, 1984).

[40] Therneau, T. M. & Atkinson, B. *rpart: Recursive Partitioning and Regression Trees*. (R Foundation for Statistical Computing, 2025). 10.32614/CRAN.package.rpart

[41] Chettri, N., Shrestha, A. B. & Sharma, E. Climate change trends and ecosystem resilience in the Hindu Kush Himalayas. In *Himalayan Weather and Climate and Their Impact on the Environment* (eds Dimri, A. P., Bookhagen, B., Stoffel, M. & Yasunari, T.) 525–552 (Springer, 2020). 10.1007/978-3-030-29684-1_25

[42] Pepin, N. et al. Elevation-dependent warming in mountain regions of the world. *Nat. Clim. Chang.* **5**, 424–430. 10.1038/nclimate2563 (2015).

[43] Ghanim, A. A. J. et al. Assessing spatiotemporal trends of total and extreme precipitation in a subtropical highland region: A climate perspective. *PLoS ONE* **18**, e0289570. 10.1371/journal.pone.0289570 (2023). 37540654 10.1371/journal.pone.0289570 PMC10403077

[44] Saleem, M., Anwar, M. W. & Ali, G. M. Analyzing the impact of ungauged hill torrents and their associated inundation streams using a quantitative methodological approach. *Resources* **12**, 26. 10.3390/resources12020026 (2023).

[45] Karki, R., Hasson, S. u., Schickhoff, U., Scholten, T. & Böhner, J. *Rising Precipitation Extremes Across Nepal*. Climate 5, 4. 10.3390/cli5010004 (2017).

[46] Lhamo, K. et al. Spatiotemporal variation of extreme precipitation indices over Bhutan (1996–2018). *Atmosphere* **14**, 286. 10.3390/atmos14020286 (2023).

[47] Krishnan, R. et al. *Assessment of Climate Change Over the Indian Region: A Report of the Ministry of Earth Sciences (MoES), Government of India* (Springer, 2020).

[48] Uddin, M. N., Bokelmann, W. & Dunn, E. S. Determinants of farmers’ perception of climate change: A case study from the coastal region of Bangladesh. *Am. J. Clim. Chang.* **6**, 151–165. 10.4236/ajcc.2017.61009 (2017).

[49] Hamilton, L. C. & Keim, B. D. Regional variation in perceptions about climate change. *Int. J. Climatol.* **29**, 2348–2352. 10.1002/joc.1930 (2009).

[50] Legesse, B., Ayele, Y. N. & Bewket, W. Smallholder farmers’ perceptions and adaptation to climate variability and climate change in Doba District, West Hararghe, Ethiopia. *Asian Journal of Empirical Research* **3**, 251–265 (2013).

[51] van der Linden, S. The social-psychological determinants of climate change risk perceptions: Towards a comprehensive model. *J. Environ. Psychol.* **41**, 112–124. 10.1016/j.jenvp.2014.11.012 (2015).

[52] Farhan, M. et al. Determinants of risk attitude and risk perception under changing climate among farmers in Punjab. *Pakistan. Natural Hazards* **114**, 2163–2176. 10.1007/s11069-022-05465-x (2022).

[53] Habtemariam, L. T., Gandorfer, M., Kassa, G. A. & Heissenhuber, A. Factors influencing smallholder farmers’ climate change perceptions: A study from farmers in Ethiopia. *Environ. Manage.* **58**, 343–358. 10.1007/s00267-016-0708-0 (2016). 27179801 10.1007/s00267-016-0708-0

[54] World Meteorological Organization. A global framework for climate services: empowering the most vulnerable. WMO High-Level Taskforce Report (2011).

[55] Tall, A. et al. Scaling up climate services for farmers: mission possible. *Learning From Good Practice in Africa and South Asia*. CCAFS Report No. 13, CGIAR Research Program on Climate Change, Agriculture and Food Security (CCAFS), (Copenhagen, 2014).

[56] Hansen, J. W. et al. Climate services can support African farmers’ context-specific adaptation needs at scale. *Front. Sustain. Food Syst.* **3**, 21. 10.3389/fsufs.2019.00021 (2019).

[57] IPCC. Summary for policymakers. In Climate change,. impacts, adaptation and vulnerability. *Contribution of Working Group II to the Sixth Assessment Report of the Intergovernmental Panel on Climate Change* 3–33 (Cambridge University Press, 2022). 10.1017/9781009325844.001

