# Supplementary material for: Do pastoral and agro-pastoral perceptions align with observed climate extremes? Evidence from the Koh-e-Suleiman Range, Pakistan
Source: Sci Rep. 2026 Mar 5;16:8275. doi: 10.1038/s41598-026-41100-6 (PMC12966273; doi:10.1038/s41598-026-41100-6)
Supplement: Supplementary file 1 — Supplementary Information. [file 41598_2026_41100_MOESM1_ESM.pdf]

Electronic Supplementary Material

This file contains additional figures and tables supporting the main manuscript.

Table S1: Location and elevation of gridded points used for spatial aggregation of meteorological data in the Koh-e-Suleiman region.

Grid ID	Latitude	Longitude	Elevation (m)
1	31.32° N	70.53° E	195.8
2	30.87° N	70.53° E	209.6
3	30.42° N	70.07° E	1169.3
4	30.42° N	70.53° E	246.1
5	29.96° N	70.07° E	1370.8
6	29.51° N	69.62° E	723.8
7	29.51° N	70.07° E	169.4
8	29.05° N	69.62° E	647.9
9	29.05° N	70.07° E	110.1
10	28.60° N	69.62° E	136.8

Note: Coordinates and elevations of 10 grid points used to extract and average climate data across the Koh-e-Suleiman region.

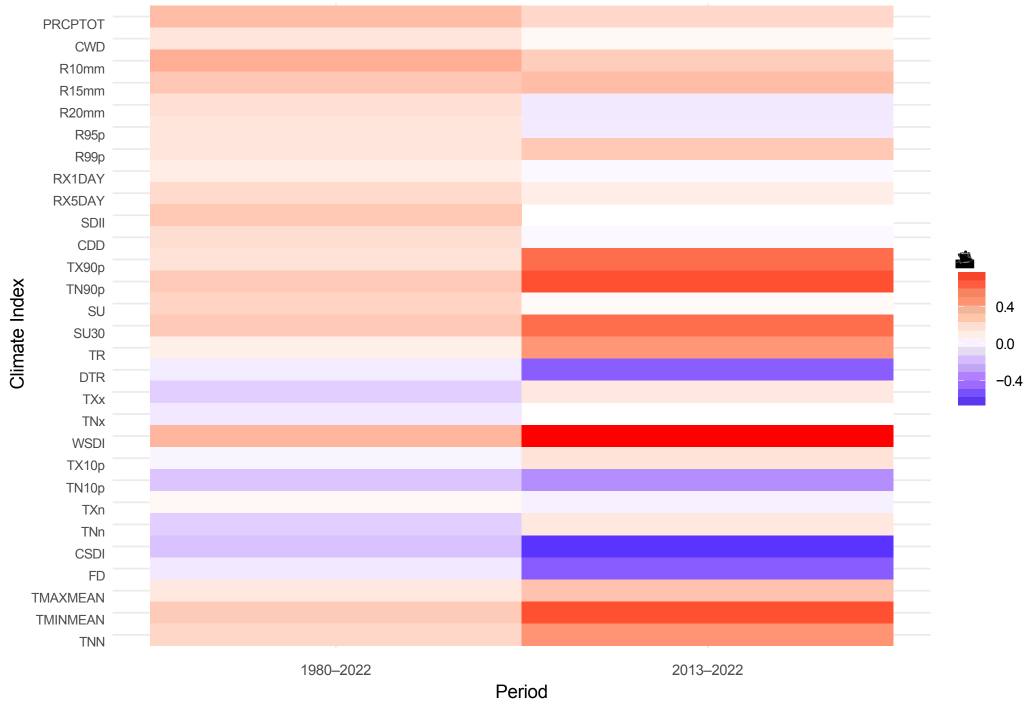

Figure S1: Mann–Kendall heat map of ETCCDI indices across two periods (1980–2022 vs. 2013–2022). Brighter warm tones in the 2013–2022 panel (e.g., WSDI, TX90p, TN90p, TMAXMEAN, TMINMEAN) indicate intensi- fied warming and more frequent warm extremes, alongside deeper cool tones for cold-extreme indices (e.g., CSDI, TNn, TXn) showing stronger declines in cold spells. Precipitation indices (e.g., PRCPTOT, Rx1day, Rx5day, SDII) show smaller but directionally consistent changes, with some increases in in- tensity in the recent period. The color scale (T) represents Mann–Kendall Z scores (trend strength), not absolute magnitudes.

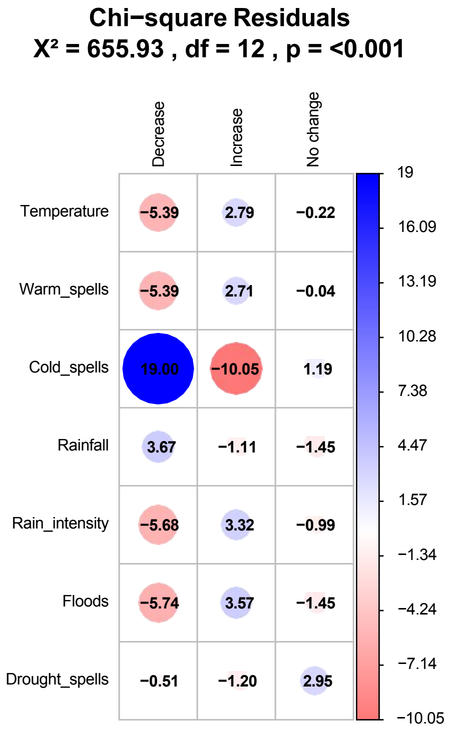

Figure S2: Chi-square residuals plot showing the degree of mismatch be- tween perceived and observed climate variables. Positive residuals indicate overestimation (e.g., drought spells), while negative residuals indicate under-estimation. The X^2 value (655.93, df = 12, $p < 0.001$) confirms significant divergence across categories.

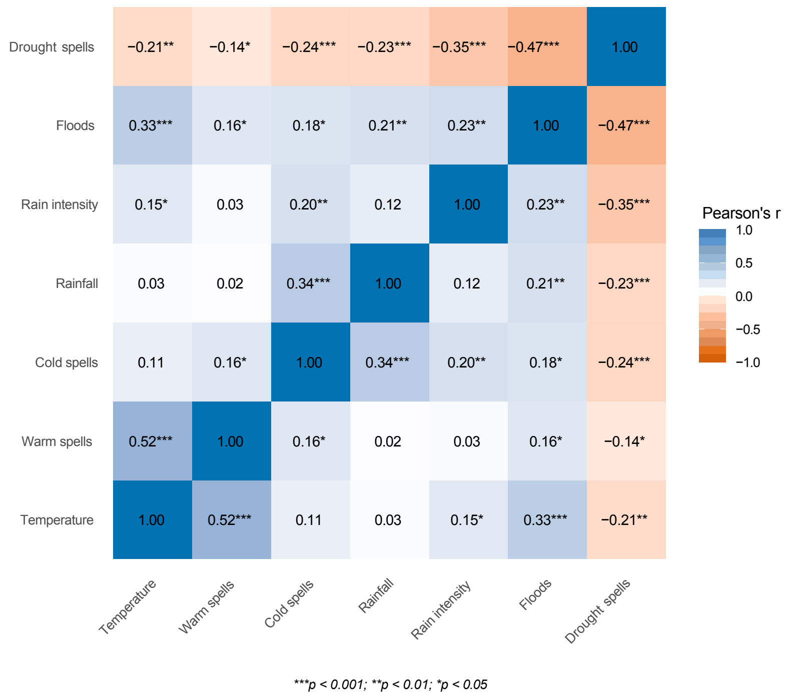

Figure S3: Correlation heatmap of perception variables. Pearson's r values indicate associations between perceptions of temperature, warm spells, cold spells, rainfall, rain intensity, floods, and drought spells. Positive correlations (red tones) highlight clusters such as temperature with warm spells, while negative correlations (blue tones) are most pronounced between drought spells and other variables. Significance levels: $***p < 0.001$, $**p < 0.01$, $*p < 0.05$.

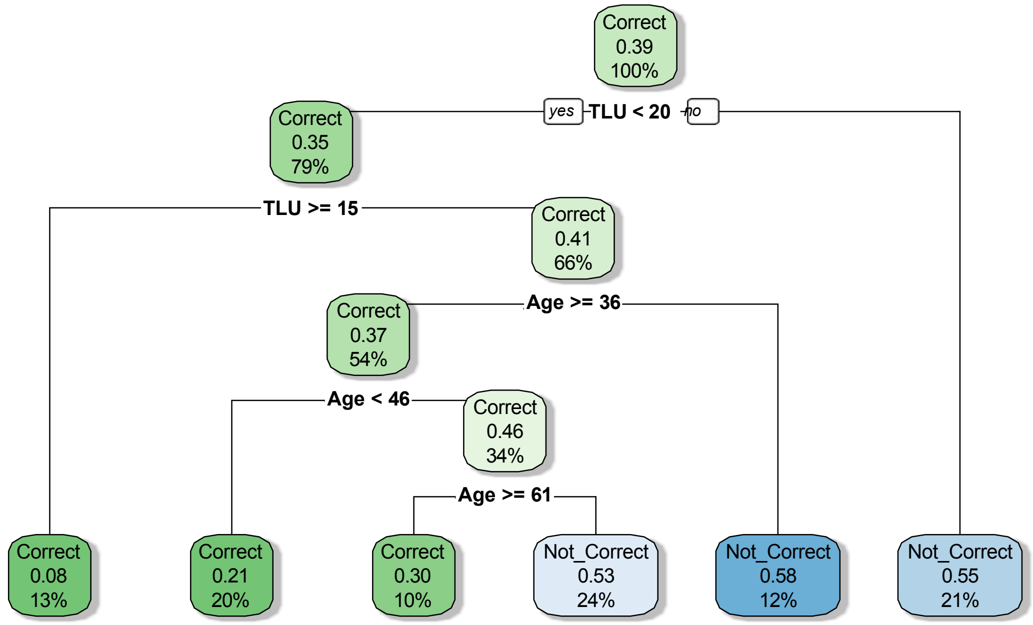

Figure S4: Classification and regression tree (CART) model predicting correct perception of rainfall changes. The decision tree identifies livestock holdings (TLU) and age as the main predictors. Terminal nodes report the proportions of respondents classified as correct versus not correct. The analysis shows that farmers with smaller herds (TLU ¡ 20) and older respondents (age ≥ 61) were more likely to perceive rainfall variability accurately, while younger individuals with larger herds exhibited higher rates of incorrect perception.

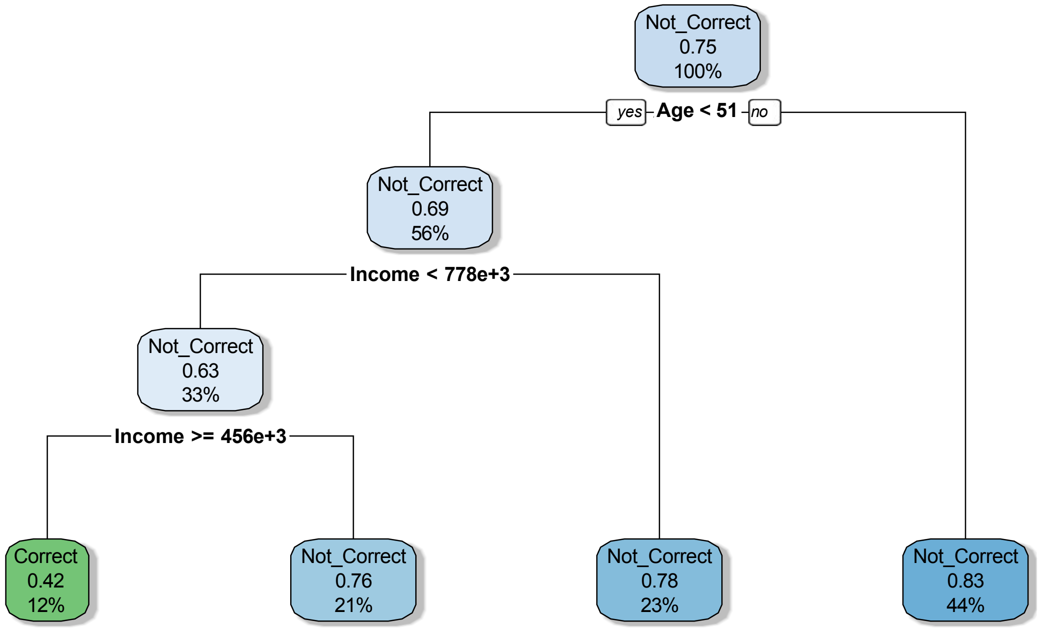

Figure S5: Classification and regression tree (CART) model predicting cor- rect perception of drought spells. The decision tree splits respondents by socio-demographic characteristics (age and income), with terminal nodes showing classification outcomes and proportions of correct versus incorrect perception. The model highlights that younger respondents with lower in- come levels were less likely to perceive drought correctly, whereas a small subgroup of older, higher-income individuals demonstrated more accurate recognition.
